# A Wireless Network for Monitoring Pesticides in Groundwater: An Inclusive Approach for a Vulnerable Kenyan Population

**DOI:** 10.3390/s24144665

**Published:** 2024-07-18

**Authors:** Titus Mutunga, Sinan Sinanovic, Colin Harrison

**Affiliations:** School of Engineering and Built Environment, Glasgow Caledonian University, Glasgow G4 0BA, Scotland, UK; sinan.sinanovic@gcu.ac.uk (S.S.); colin.harrison@gcu.ac.uk (C.H.)

**Keywords:** LoRa, water, pesticides, bulk SMS, IoT, RFID, monitoring

## Abstract

Safe drinking water is essential to a healthy lifestyle and has been recognised as a human right by numerous countries. However, the realisation of this right remains largely aspirational, particularly in impoverished nations that lack adequate resources for water quality testing. Kenya, a Sub-Saharan country, bears the brunt of this challenge. Pesticide imports in Kenya increased by 144% from 2015 to 2018, with sales data indicating that 76% of these pesticides are classified as highly hazardous. This trend continues to rise. Over 70% of Kenya’s population resides in rural areas, with 75% of the rural population engaged in agriculture and using pesticides. Agriculture is the country’s main economic activity, contributing over 30% of its gross domestic product (GDP). The situation is further exacerbated by the lack of monitoring for pesticide residues in surface water and groundwater, coupled with the absence of piped water infrastructure in rural areas. Consequently, contamination levels are high, as agricultural runoff is a major contaminant of surface water and groundwater. The increased use of pesticides to enhance agricultural productivity exacerbates environmental degradation and harms water ecosystems, adversely affecting public health. This study proposes the development of a wireless sensor system that utilizes radio-frequency identification (RFID), Long-range (LoRa) protocol and a global system for mobile communications (GSM) for monitoring pesticide prevalence in groundwater sources. From the system design, individuals with limited literacy skills, advanced age, or non-expert users can utilize it with ease. The reliability of the LoRa protocol in transmitting data packets is thoroughly investigated to ensure effective communication. The system features a user-friendly interface for straightforward data input and facilitates broader access to information by employing various remote wireless sensing methods.

## 1. Introduction

Water constitutes a critical natural resource, whose utility spans essential functions such as agriculture, industry, and domestic affairs. Access to clean safe water remains an important goal to all nations, as enshrined in UN Sustainable Development Goal no 6 [1,2]. Across nations, established water standards by regulatory bodies serve to preserve the integrity and safety of drinking water. The availability of clean water is crucial for the settlement and advancement of communities, offering numerous health, economic, and environmental benefits [3]. Water makes up approximately 60% of our body weight, facilitating various bodily functions, such as nutrient distribution, toxin elimination, and temperature regulation. Adequate water intake supports kidney and liver function, prevents digestive issues, reduces dehydration-related headaches, and maintains skin health and appearance [4]. Economically, clean water resources are vital for agriculture, hydroelectric power generation, livestock production, industry, forestry, and fisheries [4,5]. Access to clean water also impacts social well-being; for instance, a reliable water supply can significantly reduce the nearly 5000 daily deaths of children worldwide from diarrhoea-related diseases [6,7]. Additionally, it enhances the quality of life for the elderly, lowering depression levels and improving overall health [8]. Environmentally, maintaining clean river water supports aquatic life and can be used for irrigation and aquaculture. Protected ecosystems serve as venues for recreation, education, and research, promoting biodiversity and community well-being [9]. Moreover, clean rivers offer therapeutic benefits without harmful side effects by containing beneficial plant compounds like apiin and hyperoside, which have shown potential in treating conditions such as psoriasis [10]. This underscores the importance of preserving river ecosystems not just for their ecological value but also for their potential health benefits. Natural processes like geological and microbial activity as well as human activities contribute to the pollution of surface and groundwater sources [11,12]. Potential sources of water degradation include industrial effluents, agricultural runoffs, raw sewage, the presence of heavy metals, microplastics, pharmaceuticals, and other engineered materials. Pesticides have emerged as one of the major water contaminants due to their deliberate use in arable land for agricultural productivity [13].

The use of pesticides in Kenya saw a significant rise from 2015 to 2018. According to data from the Agro Chemical Association of Kenya, pesticide imports nearly tripled from 6400 tonnes to 15,600 tonnes during this period [14]. This surge, driven by farmers’ efforts to protect crops from pests and diseases, has occurred without comprehensive statistics on the impacts on humans, animals, or the environment. Alarmingly, Kenya does not monitor pesticide residues in surface and groundwater sources, despite the rising pesticide imports [15]. Sarkar et al. (2021) [15] revealed that a significant portion of pesticides used in Kenya is imported from China (42%) and the European Union (30%), and of the total sales volume, 76% of these pesticides contain one or more active ingredients classified as Highly Hazardous Pesticides (HHPs). HHPs are known for their acute and chronic toxicity, inclusion in international pesticide regulation conventions, and documented severe adverse effects on human health and the environment [16]. The Kenyan Pest Control Products Board (PCPB) has registered 247 active ingredients for use in the country; however, while the European market approves 150 of these ingredients, it has banned 78, considering them potentially dangerous [14]. Although these pesticides are banned in EU member states, certain countries still export them, exploiting Kenya’s weak policy framework and porous borders, which facilitate the smuggling of banned products into the market [17].

Glyphosate and glyphosate-based herbicides (GBHs) have been shown to induce cytotoxic and genotoxic effects in mammalian lymphocytes, particularly at high doses, alongside oxidative stress. These substances also provoke inflammation, alter cytokine production in various organs, and affect lymphocyte responses and interactions with pathogens. Human exposure to glyphosate and GBHs has been associated with genotoxic effects, disruptions in energy metabolism, interactions with gut microbiota, and the potential development of inflammatory diseases like asthma and endometriosis, necessitating further research to clarify their overall safety [18,19]. Other lethal pesticides banned in the EU but still used in Kenyan farms include atrazine (Syngenta), fipronil (BASF), chlorpyrifos (Corteva Agriscience and DowDuPont), diazinon (Adama Agricultural Solutions), and trichlorfon (Bayer). In 2018, 43% of pesticides used in Kenya were highly hazardous, and by 2021, 70% were toxic to fish [14]. Additionally, Kenya is 1 of 14 countries identified in the 2017 European Food Safety Authority (EFSA) report, published in 2019, where Maximum Residue Limits (MRLs) were exceeded in over 10% of samples tested, with residue levels surpassing European MRLs in kale, tomatoes, and water [20].

Agriculture is Kenya’s main economic activity, contributing over 30% of the GDP [21]. It is estimated that over 75% of the population is directly or indirectly involved in agricultural activities, with more than 70% of Kenya’s 53 million people living in rural areas where these activities are concentrated [22,23]. Subsistence farming in rural areas is often carried out by women, who generally have lower literacy levels than men [24]. This lack of literacy hinders their ability to read safety labels and interpret instruction manuals, leading to the misuse of pesticides [15]. A study conducted on large-scale farms in western Kenya found that 53.9% of farmers were unaware of the health and environmental impacts of pesticides [25]. The use of child labour in the agricultural sector further exacerbates the issue, as children are particularly vulnerable to the harmful effects of pesticides due to their developing nervous systems and lack of cognitive skills to follow essential safety procedures. Poisoning cases have been reported at Kenyatta National and Referral Hospital, with about 50% attributed to pesticides [14].

Water scarcity remains a pressing issue in Kenya’s rural areas, where piped water systems are absent. Approximately 41.5% of the rural population relies on shallow wells and surface water sources (lakes, streams, and ponds) for domestic water needs [26]. These water sources are situated in heavily agricultural zones, where pesticide contamination is prevalent due to widespread use for crop protection. Climate change has exacerbated the situation by altering rainfall patterns and increasing pressure on already-stressed water sources due to irrigation demands. Research indicates that the agricultural and industrial sectors contribute to 80% of water pollution and contamination in the country, with certain pesticides persisting in the environment for extended periods, ultimately contaminating water sources. The consumption of contaminated water poses significant health risks, which vary depending on the toxicity of the contaminants and the level of exposure. Several negative health impacts of water pollutants have been identified, some of which cause life-threatening conditions like cancer; hence, proper measures should be taken to safeguard vulnerable populations including pregnant women and children who are highly susceptible [14,27,28,29]. It is, therefore, imperative to frequently monitor water sources to ensure they are safe for public use. There are various methods used to detect pesticides in water. Analytical methods, also referred to as traditional, involve lab procedures and sample pre-processing stages, leading to a high demand for trained manpower and time consumption [30,31,32]. Rapid methods of detection including spectroscopic, Raman or the use of biosensors have been suggested in several studies to shorten the time of detections and, hence, qualify them as potential for point-of-care testing. In addition, the development of portable devices and the simplicity of procedures involved in these rapid methods can enable people with limited knowledge to utilize them in field testing [33,34,35,36]. The implementation of wireless sensing facilitates efficient data transmission, enabling the generation of actionable insights.

This study focuses on designing a comprehensive system for pesticide testing in regions where water sources are highly distributed, with a specific emphasis on addressing the critical issue of water contamination by pesticides in Kenya. The proposed system’s aim is to implement an innovative and cost-effective monitoring framework to detect pesticide levels in water sources. Furthermore, it seeks to ensure that the resultant information is effectively disseminated to the local population, particularly targeting vulnerable and marginalized groups, such as individuals with limited literacy, the elderly, and the economically disadvantaged. Notably, previous studies predominantly centre on centralized systems, wherein water collection occurs at a central point, followed by distribution to various regions. These investigations, thus far, have neglected to consider the imperative requirement for in situ water testing at distributed water sources, which serves as the foundational premise for this study. This case inevitably demands a decentralized testing and analysis solution. In areas exhibiting dispersed settlement patterns and devoid of a tap water facility, the applicability of a centralized water testing system becomes untenable. The spatial distribution of these sources poses a challenge for the establishment of a singular testing point. Attempts to establish routine sampling regimes and testing across those geographical locations are financially burdensome and, therefore, unattainable. Additionally, marginal areas have poor infrastructure and logistically difficult-to-collect samples for transport to centralized testing centres.

This study proposes a decentralized system for collecting pesticide data, transmitting them, analysing them, storing them, and providing feedback to users regarding pesticide detection in water. The system employs a wireless architecture that leverages various wireless technologies to design a point-of-care testing (POCT) solution for detecting pesticides in water. The system utilizes low-cost methods suitable for distributed water sources, making it accessible and practical for widespread use. Additionally, it takes advantage of bulk SMS and vernacular languages to ensure broader access to information for vulnerable groups in society. Global System for Mobile Communications (GSM) cellular networks are particularly valuable for this purpose due to their widespread coverage and ability to handle voice and short message services (SMSs) [37]. As of the end of 2023, GSM coverage reached 97% of the world [38]. Although some countries are transitioning from 2G and 3G to newer technologies like 5G and 6G, GSM’s persistence in rural areas makes SMS a reliable choice for alerts and warnings. Over three-quarters of the world population owns a mobile phone, enabling widespread access to GSM services [38]. Some parts of the rural Kenya have coverage gaps, and to enhance the communication reliability of the proposed design, the LoRa protocol is employed to relay the test results to a local server. LoRa is a low-power wireless communication protocol, with a range of up to 15 km, operating on unlicensed industrial, scientific, and medical (ISM) bands [39]. This makes it suitable for IoT ecosystems where long distances are encountered in large-scale outdoor networks. LoRa’s physical layer enables long-range communication, while the LoRaWAN media access control (MAC) layer defines the system architecture and communication protocol. Although standards like Bluetooth and Wi-Fi offer better data rates, their short-range nature limits their application in monitoring activities spanning several kilometres [40]. LoRa uses chirp spread spectrum (CSS) modulation, where data are encoded onto chirp signals [41]. The spreading factor (SF) values range from 6 to 12, with higher SFs providing better noise and interference resistance but reduced spectral efficiency [39]. LoRa nodes in a LoRaWAN network are asynchronous and use Aloha and Adaptive Data Rate (ADR) for power saving [42]. In contrast, cellular IoT standards like narrowband IoT (NB-IoT) and LTE machine-type communication (LTE-M) require frequent synchronization, leading to higher power consumption [43].

Limited literacy levels and advanced age can potentially hinder the use and acceptance of devices demanding high cognitive skills for their operation. To take care of these vulnerable groups in society, RFID is employed to capture pesticide data from the water sources. Radio-frequency identification (RFID) technology is central to Internet of Things (IoT) monitoring scenarios because of its wireless nature. RFID consists of two main components: the reader and the tag [44,45]. An RFID reader emits a continuous RF signal to interrogate a transponder, which then modulates and scatters the signal back with encoded data [45]. Passive tags, which harvest RF energy, are commonly used in monitoring due to their power efficiency. Active tags, though bulkier and more expensive, have internal batteries that provide longer read ranges and data logging capabilities. Computational RFID, which integrates circuits like analogue-to-digital converters and microcontrollers, offers accurate results, environmental immunity, and larger memory [45]. In large deployments, multiple RFID readers connected to the internet can interrogate transponders, though shared communication channels can lead to data collisions. Techniques like RF-carrier reuse and load modulation minimize such data degradation [46].

## 2. Related Work

Xiao et al. [47] monitored water quality in Tiyanik Lake using LoRa technology, achieving a 100% communication success rate within 400 m, which dropped by 25% at distances between 1.6 and 2 kilometres. They used the Open Cloud Platform (OneNET) for real-time and historical data visualization and software alerts when threshold values were exceeded. Kombo et al. [48] monitored groundwater levels in Zanzibar using a Dragino LG01-P gateway and an Arduino Uno R3. Their setup included an SD card, real-time clock, and pressure sensors for water depth measurement, with data logged every 6 h and transmitted every 12 h. Fuentes et al. [49] developed a LoRa-based water quality assessment network for fish farms, monitoring parameters, like temperature, pH, dissolved oxygen, and conductivity. The system featured a centralized probe, a gateway for data visualization, and a Raspberry Pi as the local server but faced issues with response time and material costs. Reference [50] reported on a system monitoring Lake Tunghai’s water quality using IoT and LoRaWAN, with data visualized via Grafana. They noted challenges in system design and synchronization with alarm systems. Ullah et al. [51] created an SMS-based system for regulating water levels in agricultural tubes, allowing motor activation and parameter status queries via SMS. Ugwu et al. [52] implemented a similar system for monitoring water levels in irrigation tanks using an ultrasonic sensor and SIM900 GSM module, providing SMS alerts and controlling irrigation pumps based on soil moisture and temperature. Akwu et al. [53] developed an Arduino Uno-based system for soil moisture monitoring and automatic irrigation, with SMS alerts and real-time updates via an LCD. Chafa et al. [54] used an ESP8266 Wi-Fi module to transmit sensor data on water parameters to ThingSpeak, integrating pH regulation via controlled pump activation. Nyaga et al. [55] controlled greenhouse conditions by reading sensor data and relaying them to users via Bluetooth or SMS, with an LCD for data display.

It is notable from the above studies that the LoRa communication protocol is typically utilized for packet transmission over shorter distances, in contrast to GSM, which facilitates global message transmission. Due to cost implications, GSM is more suitable for transmitting synthesized information, such as alerts and notifications. Although the studies have demonstrated various applications of wireless sensing, none have specifically addressed the needs of vulnerable and marginalized members of society, such as individuals with limited literacy, the elderly, or the economically disadvantaged. This current research addresses these gaps by considering these groups. Presenting information to the public in an easily understandable language can significantly enhance its impact and elicit desirable responses. The public comprises a diverse mix of individuals, many of whom may lack the knowledge required to interpret data gathered by sensors.

## 3. Materials and Methods

### 3.1. System Architecture

**Figure 1:**
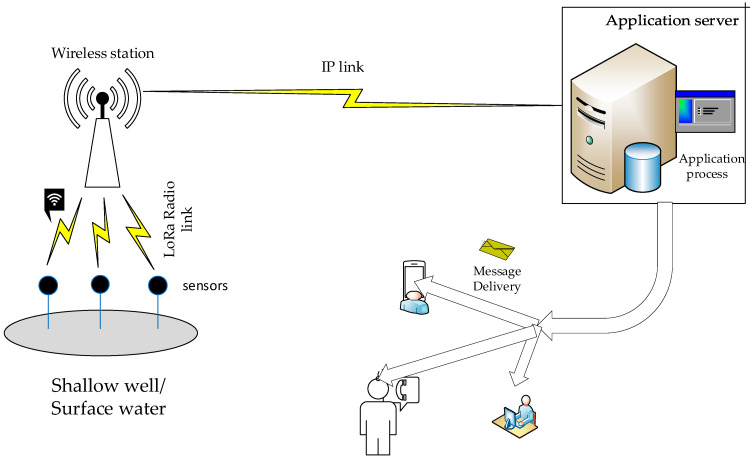
Proposed architecture of wireless sensor network.

A codesign of hardware and software is suggested, though performed separately, to enable greater flexibility in the adaptation of the partitions and give room for thorough troubleshooting [56]. The sensor and the server are designed with Arduino Uno and several compatible shields. Table 1 provides a list of the components utilized in the design. These form the main hardware employed while the user interface with controls for data capture, data communication, analysis, and decision making is implemented using the Arduino open-source IDE. Various wireless communication links applied in the system are given in Figure 1.

The deployment scenario involves various devices, with sensors placed in the field for data gathering. The network topology employs the LoRa protocol for data transmission from the field, which is advantageous because it allows data to be sent from remote areas over distances exceeding 10 kilometres without requiring mobile coverage [58]. This is particularly useful for monitoring wells in rural areas, especially in riparian zones where mobile coverage is often lacking. These zones are common locations for wells, and the LoRa protocol effectively addresses the challenge of connectivity gaps in such remote areas. Packets sent by LoRa nodes are picked up by a LoRa Gateway and then forwarded to a server for processing. The server checks the condition of the received packet. If the packet positively identifies the presence of pesticides from the field, bulk SMS notifications alerting the registered users are sent. Otherwise, messages of conformity are distributed to the registered members. Detailed information is available in a portal for any member with access to view the condition of the field.

### 3.2. The Sensing Unit

**Figure 2:**
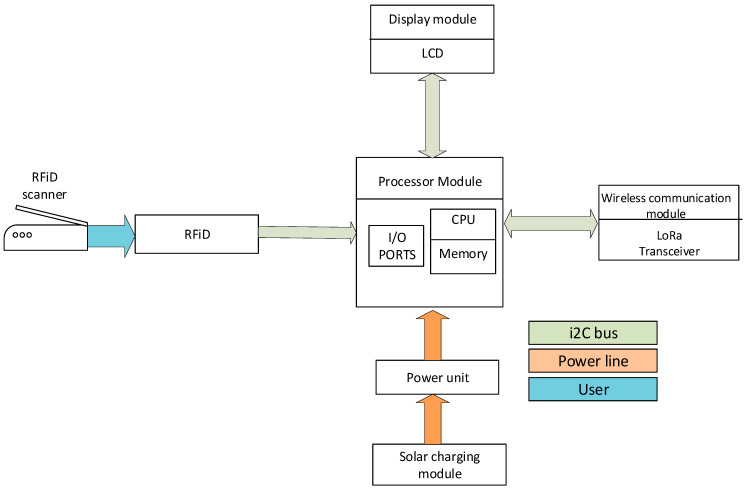
Block diagram of proposed sensing module.

The main control chip adopts ATmega328 by Atmel. Arduino Uno consists of I2C support pins, ADC, SPI, and PWM as well as transmit–receive pins. Additionally, it includes an in-built 16 KB flash memory, 2 KB SRAM, and 1 KB EEPROM. Three shields are stacked on board the RFID, LCD Keypad, and LoRa transceiver. An RFID card reader is used as the main input to the sensor. To ensure a step-wise flow of procedures, an LCD keypad provides prompts to the user and an environment to input data. Figure 2 illustrates the various modules making up the sensor.

Paper-based sensors were selected for this study due to their colourimetric nature, which allows for easy readout with the naked eye, without the need for sophisticated instruments. These sensors are also more cost-effective compared to other testing methods [59]. Various paper-based sensors were identified for determining different pesticides. The sensor used in this study is an immunochromatographic lateral flow device, specifically the Abraxis glyphosate strip by Gold Standards Diagnostics (Warminster, PA, USA). This sensor includes a window where colour changes can be observed upon the addition of a pesticide. The window is coated with antibodies that bind to antigens on the pad. A control line appears to indicate a valid result. Sample concentrations are assessed by comparing the intensity of the test line to that of the control line on the same strip. For results to be deemed valid, a visible control line must be present, regardless of its intensity [60]. If the test line is darker than or matches the control line, the result is below the test’s detection limit. If the test line is lighter than the control line, it signifies a low to moderate concentration. A very faint test line or the absence of a test line indicates a high concentration. The FSTest, another paper-based test strip developed for the screening of organophosphate and carbamate pesticides, operates based on the inhibition effects against acetylcholinesterase (AChE) activity. Pesticides in the sample exhibit an inhibitory effect on AChE, which catalyses the hydrolysis of indophenol acetate to produce acetic acid and indophenol. Consequently, the absence of or a reduction in the blue colour correlates with the pesticide concentration in the samples. The test strips are designed to be foldable, consisting of red and white zones. The sample is placed on the white zone, and the paper is then folded to bring the two zones into contact. Upon unfolding, the intensity of the blue colour formed on the previously white zone indicates the concentration of pesticides, with a faded blue colour signifying a higher concentration. Pesticide residue meters provide quantitative results by measuring the absorbance levels of coloured by-products resulting from biochemical reactions. In one such meter, cholinesterase catalyses the hydrolysis of acetylcholine in the presence of DTNB (5,5’-dithiobis (2-nitrobenzoic acid)), producing a yellow-coloured compound, 5-thio-2-nitrobenzoic acid (TNB). The presence of organophosphate pesticides inhibits cholinesterase activity, leading to reduced hydrolysis of acetylcholine and, consequently, less yellow colour formation. The degree of yellow colouration, measured as absorbance, correlates with the activity of cholinesterase. A lower absorbance indicates greater inhibition and, thus, a higher concentration of pesticide residues.

**Figure 3:**
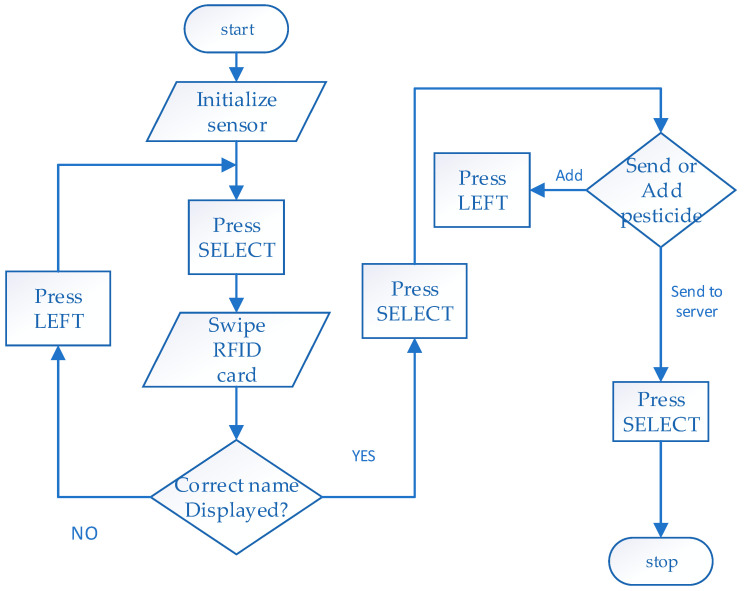
Flow chart of data capture using the input module.

The chart in Figure 3 below illustrates the steps of feeding data to the sensor. With the power on, the sensor goes through the boot procedure, initializing the different modules. The swiping of an RFID card on the device prints the name of the designated pesticide on the screen. The user is then prompted to enter their observation from the test performed. The system provides flexibility for data entry, allowing for deletions and multiple inputs until the user is ready to send the data. If a wrong entry is made, the user can cancel it by pressing the LEFT button. Additionally, if the user performs multiple tests, the system can capture all the test data as a single packet for transmission.

### 3.3. User Interface (UI) and Interaction

The operational protocol entails the initiation of normal procedures via user intervention to capture sensor data from a paper-based sensor. This is achieved through the actuation of the select button on the keypad interface, prompting the sensor to prepare for user input per the available menu options. The system provides an interactive process by actively engaging the user throughout the data-capturing process.

One of the important design aspects of the interface is to ensure simplicity when applied in the field of non-expert users of technology. After the boot procedure is complete, a query window appears on display, prompting the user to swipe the chemical RFID tag card. The swiping captures the chemical name in the internal registers. The user is then given an option to undo the activity if the wrong card is swiped. The menu then proceeds to allow the user to save the data. In case the user intends to test several pesticides, then they are allowed to do so until all the tests are over, after which they are directed to send to the server. The inputs from the users are reduced to selection using push buttons. The software gives prompts to the user on the display for the next step until data are well captured and sent. The RFID and LCD keypad shields facilitate those functions.

Pesticides have potentially complicated naming taxonomy that may predispose users to typographical errors if reliant on manual keypad entry. Controlling such occurrences is essential for ensuring data integrity and system efficiency. The adoption of radio-frequency identification (RFID) cards, preloaded with accurate pesticide names, presents a proactive measure to limit potential typographical inaccuracies. The process substantially diminishes the probability of transcription errors occasioned by manual typing. Consequently, users can swiftly capture and transmit pesticide data to the server for processing, thus expediting data acquisition workflows. Moreover, the technology promotes efficiency by simplifying complications associated with data entry. As a result, the turnaround time is reduced, a factor attributable to the minimization of cognitive load and the operational complexities for the users.

Restricting the number of exposed push buttons to just two serves as a deliberate mechanism to mitigate inadvertent button activations, thereby averting erroneous button inputs. This design choice is instrumental in ensuring that user engagement remains focused and aligned with the intended interactions. Furthermore, the customization of the select button functionality to enable data capture synchronously with the sequential progression of menu iterations represents a simplified approach to streamlining user engagement processes. This user-centric approach holds promise to enhance usability, particularly by those with advanced age or lower literacy levels. The described methodology allows for efficient data capture with minimal reliance on on-screen visual feedback cues, presenting a pragmatic solution to address potential cognitive barriers.

To further address the requirements of individuals with limited literacy levels within society, software programs are systematically engineered to employ simplified language patterns localized to the users’ indigenous dialects. By condensing messaging content and minimizing verbosity, potential comprehension barriers are checked, thereby reducing the likelihood of misinterpretation and consequent erroneous actions. The strategic selection of widely known terms utilizing vernacular expressions familiar to the populace directly conveys vital information about wells. This inclusive measure strengthens the adaptability of the system for deployment within communities characterized by constrained educational backgrounds. Integration of the local language is a key measure to eliminate the requirement of intermediary translation services. Consequently, the dissemination of critical information is efficiently performed, fostering heightened community awareness and facilitating a swifter communication process. The utilization of indigenous dialects not only enhances understanding but amplifies engagement levels, reaching broader societal participation and boosting information propagation.

### 3.4. The Server 

**Figure 4:**
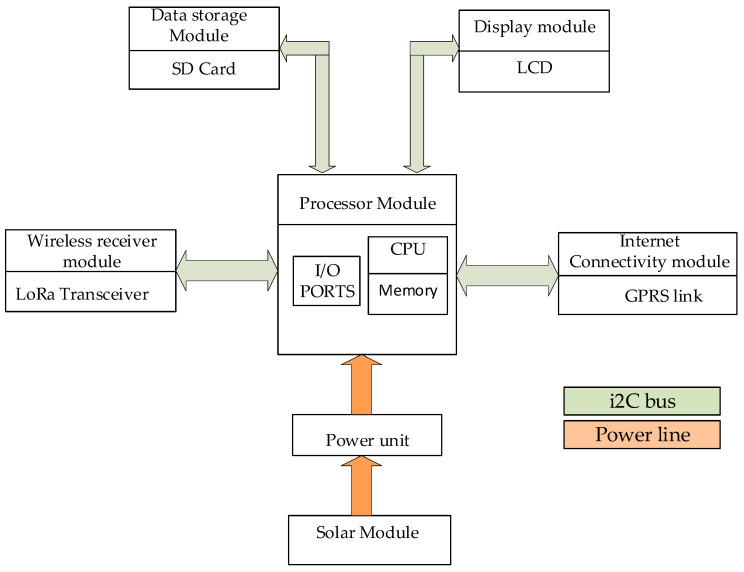
Block diagram of the proposed server module.

The server is based on an Arduino R4, featuring a 32-bit microcontroller from the RA4M1 series by Renesas (model R7FA4M1AB3CFM#AA0) and an ESP32-S3 Wi-Fi module (model ESP32-S3-MINI-1-N8). The ESP32-S3 module includes an Xtensa dual-core 32-bit LX7 microcontroller with built-in antennas for Wi-Fi and Bluetooth connectivity. The RA4M1 microcontroller is based on a 48 MHz Arm Cortex-M4 processor, and the board provides 256 KB of flash memory, 32 KB of SRAM, and 8 KB of EEPROM. The Arduino R4 board supports multiple communication protocols, including UART, SPI, I2C, and CAN. Additionally, it is equipped with a 14-bit ADC, a 12-bit DAC, and an operational amplifier. The server consists of a LoRa transceiver to facilitate the receiving of packets sent from the field. Using the ESP32-S3 Wi-Fi module, the server links to the internet to update the portal for incoming packets. The portal refreshes with each incoming packet from the field. The GSM console is dedicated to sending bulk SMS to the registered users after processing by the application server. An SD card is included in the setup, as indicated in Figure 4 below, as well as an LCD keypad for displaying the received packets.

**Figure 5:**
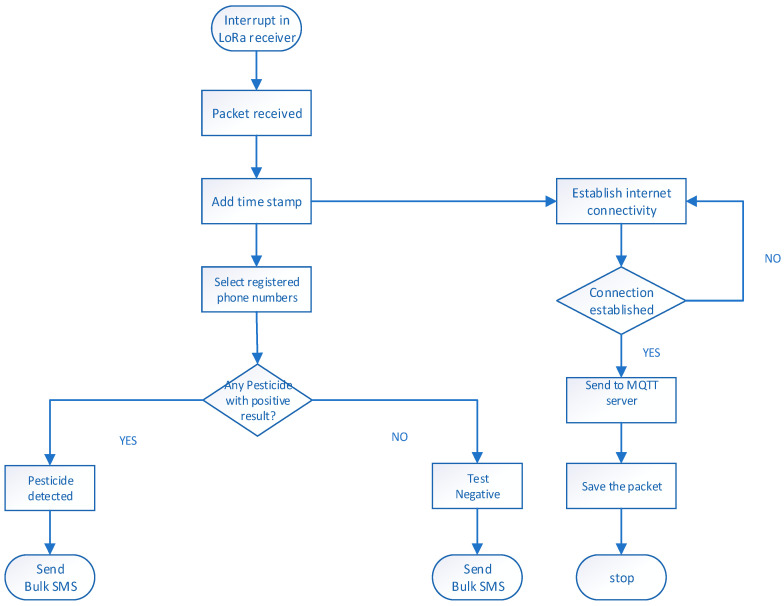
Flow chart of the server module.

User numbers are stored in the Arduino’s memory and queued for receiving test results from the field. This setup enables users to receive updates after tests are conducted on the wells. The updates are received in the form of an SMS, indicating the presence or absence of pesticides in the wells. Users can also log in to the portal to access detailed test results from an online platform. Once the server receives a packet from a node in the field, as illustrated in Figure 5, it adds a time stamp and then verifies the origin of the packet as well as the corresponding well users. The packets are then analysed for the presence of chemicals to determine the appropriate message. A bulk SMS loop is then activated to broadcast the messages to all the registered users about the status of the well and simultaneously publishes the received packet on the portal. Finally, the packet is stored on the SD card for record keeping.

## 4. Experiment

There exist several configurable transmission parameters that influence the network performance in LoRa communications. They include frequency, bandwidth (Bw), coding rate (*C_R_*), spreading factor (SF), and transmission power. The optimization of these parameters guarantees network resilience against interference, transmission range, and battery lifetime [61,62,63,64]. Frequency is a region-specific parameter, while other variables, including payload, can be adjusted to achieve desirable results. Experimental data have demonstrated that the packet error rate (PER), an important performance metric, tends to increase with higher bandwidth and payload and decrease with a higher spreading factor (SF) and coding rate (*C_R_*). Additionally, signal strength is dependent on transmission power [65]. The packet delivery ratio (PDR), another critical metric, decreases when the payload increases, while a high *C_R_* supports a higher PDR. Similarly, a high SF results in an elevated PDR [66].

The LoRa symbol rate is given as

(1) $$R_{s}=\frac{Bw}{2^{SF}}$$

where $R_{s}$ = symbol rate in baud

Bw = bandwidth in Hertz (Hz)

SF = spreading factor

Most programmed Bw values are 125,250 and 500 KHz, while the SF range is between 6 and 12. SF 6 is not used in LoRa communications.

The symbol duration, $T_{s}=\frac{1}{R_{s}}$

when substituted in (1) above yields

(2) $$T_{s}=\frac{2^{SF}}{Bw}$$

From (2), symbol duration increases with SF; hence, packets take longer to transmit, implying long time on air (TOA). Additionally, receiver sensitivity is enhanced by reducing the minimum required SNR for successful demodulation, resulting in an improved link budget.

A high Bw results in higher symbol rates, meaning that symbols are transmitted more frequently. This can, in turn, make packets susceptible to errors due to symbol interference and receiver processing time, negatively impacting the bit error rate (BER).

LoRa communication relies on forward error correction (FEC) for error detection and correction. A high coding rate (*C_R_*) introduces more redundancy in transmitted data by dedicating a large portion of the signal to error bits at the expense of effective data.

(3) $$C_{R}=\frac{4}{4+n},\;for\;n=0,1\ldots 4$$

When n = 4, $C_{R}=\frac{4}{8}$. Out of 8 bits in a packet, only 4 bits carry useful information, with the rest utilized for error correction, giving higher chances of error-free packet delivery.

If the probability of successful error correction is denoted by $P_{SE}\left(C_{R}\right),$

then,

(4) $$BER=1-P_{SE}\left(C_{R}\right)$$

Other factors like the signal-to-noise ratio (SNR) and interference still play a role.

In this work, we study the communication reliability of the system by evaluating the spatial behaviour of the LoRa signal. Analysed metrics include range, RSSI, SNR, and BER. The data are collected to establish the variations in the received LoRa signal with the distance. Since the system is meant to ensure the reliability of packet delivery, the following LoRa settings in Table 2 were adopted. The system relies on renewable energy by harvesting solar energy and experiences low-frequency data sampling. 

### Study Site Description

Glasgow Sighthill is chosen as the area of investigation because it is still undergoing construction, and some sections have a perimeter fence, deterring many people from accessing the area. The study site extends to neighbouring areas, including Sighthill Cemetery, Glasgow Kelvins College, and Springburn Shopping Centre.

In this study, we focus on the low-level radio communication to measure the link characteristics. All experiments adhered to ethical guidelines, ensuring minimal environmental impact and compliance with local regulations. One of the Arduino Uno labelled as the sender is fitted with an SD card shield to store the received packets from the sender. A program is written in C/C++, where the sender initiates the transmission by dispatching the initial packet with a size of ten bytes and subsequently awaits an acknowledgement from the receiver. Following the acknowledgement, the sender proceeds to dispatch an additional 49 packets with intervals of 50 milliseconds between them, without actively awaiting acknowledgement. This sequence constitutes a single iteration, denoted as a run, which is repeated two times in succession. These runs are then systematically executed twice for each preselected distance from the receiver [67,68], facilitating precise measurement procedures. The insertion of a delay between transmitted packets serves to provide the microcontroller with adequate time for writing the packets into the SD card to avoid skipping due to congestion of the packets. This is a measure to ensure the integrity of the data transmission process.

**Figure 6:**
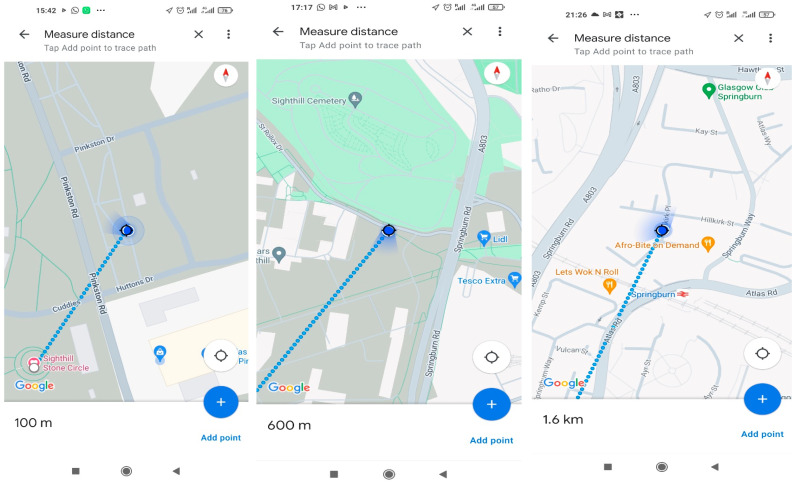
An illustration of distance measurement.

The measurement of distance for the various tests is carried out on the Google Maps platform, with the measurement of distance feature activated, as shown in Figure 6. Accuracy is further enhanced by turning on the improve location accuracy tab before making measurements.

**Figure 7:**
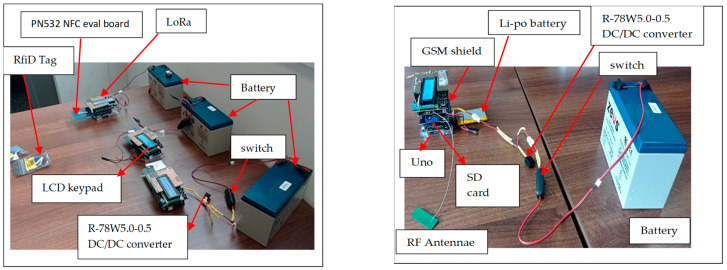
A set-up of sensors and the server.

To demonstrate the functionality of the wireless architecture before actual deployment in the field, packets were transmitted randomly from three sensors positioned at varying distances from the server, as shown in Figure 7. A mobile phone provided a hotspot for Wi-Fi connectivity to enable internet access. Additionally, three mobile devices were registered to receive the system’s feedback after processing the packets. 

## 5. Results and Discussion

**Figure 8:**
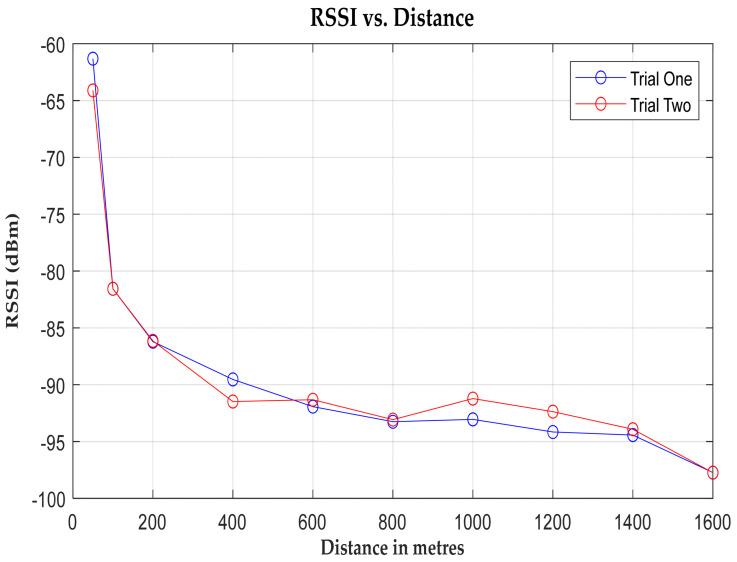
A graph of RSSI vs. distance (m).

The analysis of the Received Signal Strength Indication (RSSI) values reveals a consistent pattern: as the distance from the receiver increases, the RSSI values decrease. This decline can be attributed to signal degradation caused by atmospheric conditions, resulting in attenuation. The graph in Figure 8 visually illustrates this phenomenon. A strong signal strength of 98 dBm at 1.6 kilometres suggests that packets could potentially be received even beyond this range, were it not for the presence of tall and densely packed structures in the Springburn shopping centre, which act as barriers to signal transmission. In monitoring scenarios, where a single hop of data fails to effectively relay traffic to the desired gateway due to distance, repeater nodes can be used to pick up and retransmit the signals. When several such nodes are installed, a mesh network is created. Mesh networks are preferred in LoRa for their extensive coverage, spanning hundreds of square kilometres [69]. However, this comes at the cost of battery life and increased complexity, as nodes must relay irrelevant packets from other nodes. Therefore, a star topology is more desirable if long-range connectivity can be achieved.

**Figure 9:**
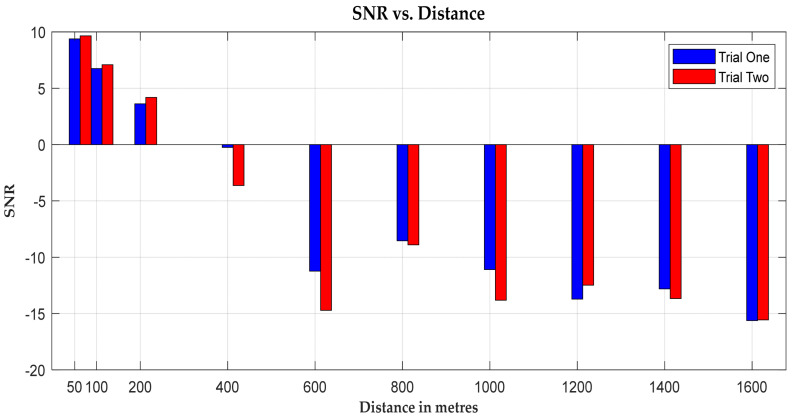
A graph of SNR vs. distance (m).

Examining the signal-to-noise ratio (SNR) in Figure 9 provides further insight. Initially, the SNR registers a positive value, indicating proximity to the transmitter. However, as the distance from the transmitter increases, the signal strength diminishes due to the increased path loss, and the SNR decreases. Notably, a significant drop in SNR occurs at the 600 m mark, corresponding to the valley of Fountainwell Road, adjacent to Sighthill Cemetery. Here, the line of sight is obstructed, leading to a reduction in signal strength.

**Figure 10:**
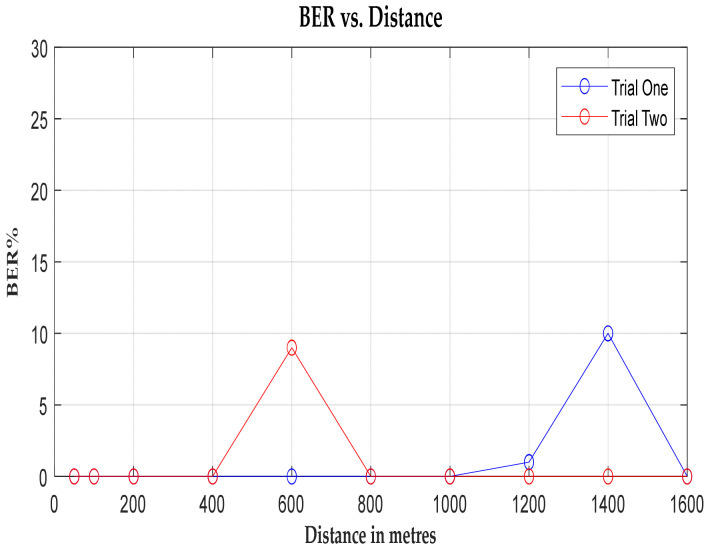
A graph of BER vs. distance (m).

**Figure 11:**
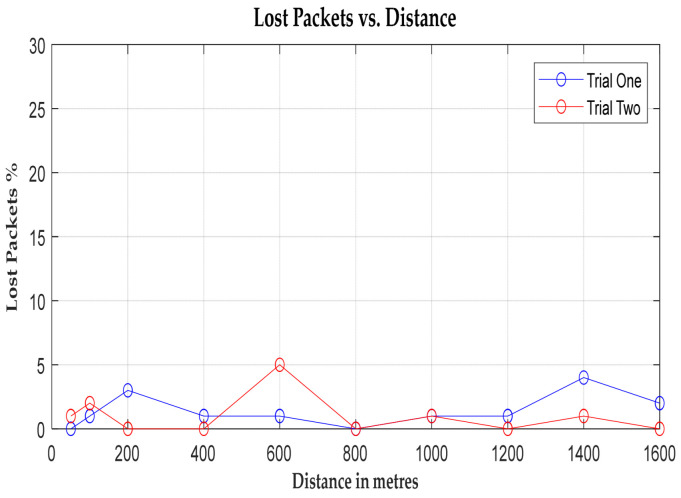
A graph of lost packets vs. distance (m).

The graphical representation unveils two prominent peaks: one at 600 m and another at 1.4 kilometres. The latter coincides with a depression at Springburn railway station, which again causes signal fading. Despite variations in signal strength and noise levels, the system demonstrates robustness for communication, with both the packet error rate (BER) and packet loss rate remaining below 10% [47] within the specified range, as illustrated in Figure 10 and Figure 11. This underscores the system’s reliability for real-world deployment.

**Figure 12:**
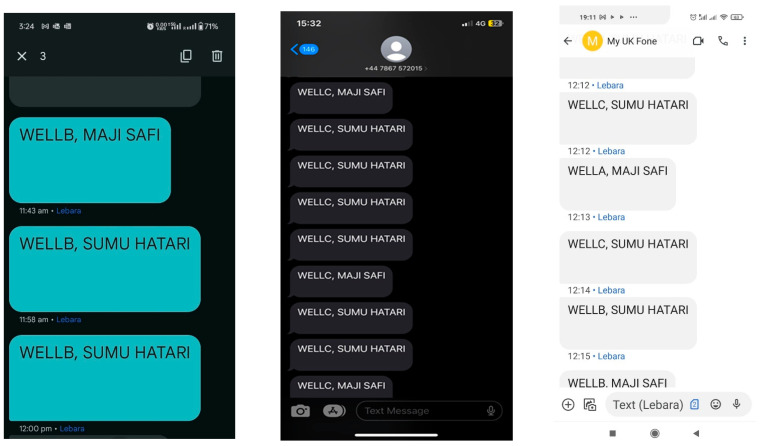
Bulk SMS for the registered users.

The local server is equipped with a GSM shield to broadcast bulk SMS to registered users after identifying the relevant members. Three handsets were used to receive the SMS notifications generated upon the receipt of LoRa packets from field sensors. Two of these handsets had their SIM cards registered to receive SMS notifications from packets sent by two different sensors, while the third handset was registered to receive messages from all sensors. Figure 12 below illustrates the SMS notifications delivered to mobile phones. It was observed that for every packet received by the server, a corresponding SMS was delivered to a designated mobile device. The portal continuously updated with the received pesticide name and the respective RSSI values. However, it was noted that during instances when the mobile phone providing the Wi-Fi connection received a phone call, the packets transmitted at that time were not published. This suggests an interruption in the Wi-Fi connection caused by the phone call. Therefore, it is recommended to utilize a dedicated router for Wi-Fi provision to avoid such interruptions and ensure continuous and reliable connectivity.

Utilizing vernacular languages for messages targeting the local population significantly enhances access and comprehension. Specifically, the Kiswahili terms “safi” (clean) and “sumu” (poison) are employed to effectively communicate the water quality status. The phrase “Maji safi”, meaning clean water, is used to inform the public that the water from a particular well is currently safe for consumption. Conversely, “Sumu Hatari”, meaning dangerous poison, serves as a warning about potential water contamination. This method ensures that individuals with limited literacy skills can easily interpret the messages and respond appropriately. Kiswahili, the national language of Kenya and widely accepted in official contexts, is familiar to the general populace. Furthermore, members registered to receive updates about a specific well receive messages exclusively from that well, ensuring they obtain only relevant information. GSM phones, being relatively inexpensive compared to smartphones, are affordable to a broader population. This ensures that crucial water quality information reaches a larger portion of the targeted demographic, thereby facilitating prompt and informed responses to water safety issues. In comparison with other commercial sensors, the proposed sensor is low cost, with the components readily available in the market, as provided in Table 3 below. The quoted price is exclusively for the equipment; other consumables are not factored into the cost of existing sensors, which may lead to an additional cost per test.

**Figure 13:**
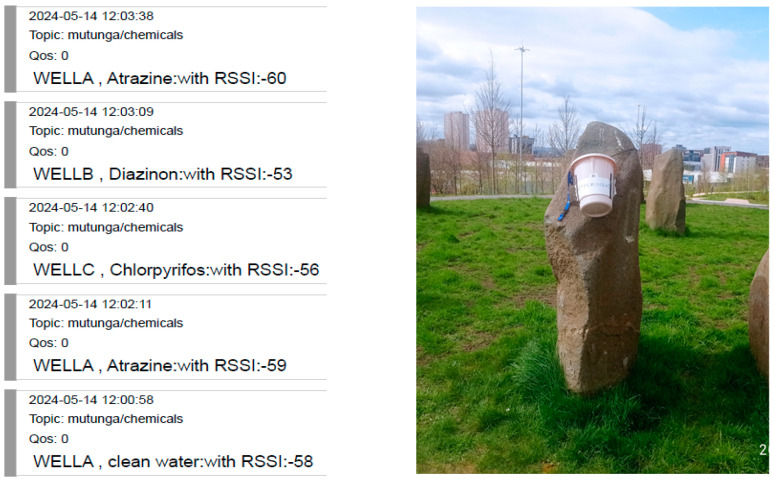
The HiveMQ portal console.

An online portal utilizing the MQTT protocol is employed for visualizing the results, as illustrated in Figure 13. MQTT is a lightweight protocol that allows constrained devices to publish messages over the internet [70]. Designed for secure networks, it uses the Internet Protocol (IP) as its transport layer. Whenever a packet is received from the field, a message is posted online using the HiveMQ broker, enabling viewers to know the status of the wells in real time, regardless of their location. The detailed information targets a diverse audience, capturing the correct names of the detected pesticides along with the specific wells where tests were conducted, as shown below. Additionally, the captured data are saved on an SD card, providing a reliable storage mechanism for the collected data. This system ensures that all stakeholders have access to up-to-date and accurate information, facilitating informed decision making and effective management of pesticide levels in water sources.

## 6. Conclusions

We have demonstrated that integrating various technologies offers an affordable and effective method for pesticide detection. Notably, this system can monitor pesticides in distributed water sources due to its low cost and simplicity. The decentralized testing, bulk SMS feedback system, and use of vernacular language enhance public access to information. The proposed use of paper strips addresses cost challenges, as they offer a more affordable testing method compared to existing alternatives. Additionally, paper strips are easy to use and interpret, significantly reducing the time required for testing. However, these tests are qualitative, making them suitable for initial screening, with further quantitative analysis needed for comprehensive results. Our observations during scanning indicated that RFID tags could be identified by an NFC reader at considerable distances, approximately 6.1 cm. Tag identification occurred, even when the tags were positioned above or below the reader, and scanning was possible despite the presence of non-metallic obstacles. Although no formal tests were conducted for RFID, these findings suggest that the sensors can be enclosed in mechanically robust casings, enhancing their durability in harsh environmental conditions. LoRa frequencies, which are free to use, enable data transmission over large geographical areas, providing an excellent opportunity for data collection from remote areas lacking power and infrastructure. A closer examination of the data suggests that signal detection is possible beyond the 1.6 km in the studied urban setup, with potentially longer ranges in rural areas. However, we observed poor reception of LoRa packets in depressions and valleys, indicating that non-line-of-sight (NLOS) conditions due to hilly landscapes can adversely affect transmission. Feedback relayed to users over the GSM network ensures wider coverage and robustness. This combination of technologies can serve as a POCT system for pesticides in water sources, providing real-time results for timely decision making and actions. Additionally, the system ensures low implementation and maintenance costs, making it a viable solution for widespread adoption.

## Figures and Tables

**Table 1:** Hardware setup.

Component	Function
Arduino Uno R3/R4 evaluation expansion board	Hosts of the microcontroller/sketches are burned here.
Fona 808 shield GSM/GPS	Hosts the GSM card/Sending SMS to users
SD card 2 GB class 6 SLC	Store the received packets from the wells
SD card shield v4 board, 3.5 v to 5.5 v, Arduino board	To host the SD card
PN532 near field communication (NFC) RF Arduino platform evaluation expansion board	Reader for RFID tags
LCD keypad shield, gravity 1602, 2 × 16 LCD, Arduino development board	To display the menu/interact with the user
12 V sealed lead acid (SLA, VRLA) battery rechargeable(secondary) 7 Ah	To power the system
R-78W5.0-0.5 DC/DC converter 5 V	Stepping voltage down from 12 V to 5 V for Arduino
RFID tag 13.56 MHz ISO14443- [57]	Loaded with pesticide names
850 MHz, 900 MHz, 1.8 GHz, 1.9 GHz, 2.1 GHz GSM, WCDMA PCB trace RF Antenna 824~960 MHz	Enhancing signal strength
rechargeable battery, 3.7 V, lithium polymer, 2 AH, JST	Power the GSM/GPS shield
LoRa/fsk transceiver module, 915 mhz, rfm97cw (com-18084)	Send or receive packets

**Table 2:** LoRa parameters.

LoRa Parameter	Value
Spreading factor	12
Coding rate	4/8
Bandwidth	125 KHz
Transmission power	20 dBm

**Table 3:** Cost comparison: proposed sensor vs. existing sensors.

Proposed Sensor	Cost	Existing Sensors	Cost
ARDUINO UNO R3 ATMEGA328P EVAL	GBP 22.26	Metrohm Misa SERS Raman	GBP 30,637.95
PN532 Near Field Communication (NFC) RF Arduino Platform Evaluation Expansion Board	GBP 32.22	Agilent 6460C QQQ Triple Quadrupole MS system with Agilent 1290 UHPLC front-end	GBP 110,760.00
RFiD tag 13.56 MHz ISO14443- [57]	GBP 1.78	Shimadzu Prominence-i LC-2030C plus HPLC	GBP 19,779.00
LCD Keypad Shield, Gravity 1602, 2 × 16	GBP 7.99	Hamamatsu OEM Raman module	GBP 7602.00
LoRa/FSK Transceiver Module, 915 MHz, RFM97CW	GBP 10.50	Raman Microscope (Mapping) ATR8300MP	GBP 35,481.00
LoRa Antenna with Pigtail—915 MHz Black	GBP 4.90	Waters Acquity UPC2 System with PDA	GBP 23,734.00
Glyphosate Dipstick-single test	GBP 21.73	Varian 920-LC, UV Varian HPLC system 920-LC	GBP 8479.00
TOTAL COST FOR PROPOSED SENSOR	GBP 101.38		

## Data Availability

The raw data supporting the conclusions of this article will be made available by the authors on request.
